# Methyl 2,6-bis­[(5-chloro-4,6-dimeth­oxy­pyrimidin-2-yl)­oxy]benzoate

**DOI:** 10.1107/S1600536810024785

**Published:** 2010-06-30

**Authors:** Hoong-Kun Fun, Jia Hao Goh, Sankappa Rai, Arun M. Isloor, Prakash Shetty

**Affiliations:** a X-ray Crystallography Unit, School of Physics, Universiti Sains Malaysia, 11800 USM, Penang, Malaysia; b Department of Chemistry, Manipal Institute of Technology, Manipal University, Manipal 576 104, India; c Organic Chemistry Division, Department of Chemistry, National Institute of Technology-Karnataka, Surathkal, Mangalore 575 025, India; d Department of Printing, Manipal Institute of Technology, Manipal University, Manipal 576 104, India

## Abstract

In the title compound, C_20_H_18_Cl_2_N_4_O_8_, the two pyrimidine rings are inclined at dihedral angles of 66.68 (5) and 71.91 (6)° with respect to the central benzene ring. In the crystal structure, inter­molecular C—H⋯N hydrogen bonds link neighbouring mol­ecules into a ribbon-like structure along the *b* axis. The ribbons are inter­connected into a two-dimensional network parallel to the *bc* plane by short inter­molecular Cl⋯Cl [3.4427 (6) Å] and Cl⋯O [3.1420 (9) and 3.1750 (11) Å] inter­actions. The crystal structure is further stabilized by inter­molecular π–π inter­actions [centroid–centroid distance 3.4552 (8) Å] involving the pyrimidine rings.

## Related literature

For general background to and applications of the title compound, see: Koichiro *et al.* (1988 ▶, 1998 ▶); He *et al.* (2007 ▶); Li *et al.* (2006 ▶); Gerorge (1983 ▶). For graph-set descriptions of hydrogen-bonded ring motifs, see: Bernstein *et al.* (1995 ▶). For a closely related structure, see: Li & Luo (2006 ▶). For the stability of the temperature controller used for the data collection, see: Cosier & Glazer (1986 ▶).
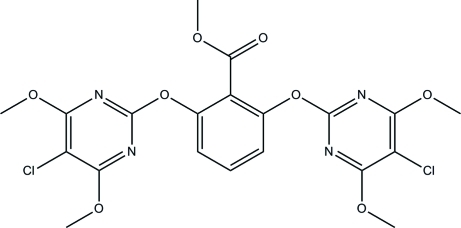


## Experimental

### 

#### Crystal data

C_20_H_18_Cl_2_N_4_O_8_
*M* *_r_* = 513.28
Monoclinic, 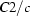

*a* = 29.354 (3) Å
*b* = 8.0485 (8) Å
*c* = 22.5923 (19) Å
β = 123.014 (2)°
*V* = 4475.7 (7) Å^3^
*Z* = 8
Mo *K*α radiation
μ = 0.35 mm^−1^
*T* = 100 K
0.58 × 0.31 × 0.16 mm

#### Data collection

Bruker APEXII DUO CCD area-detector diffractometer
Absorption correction: multi-scan (*SADABS*; Bruker, 2009 ▶) *T*_min_ = 0.825, *T*_max_ = 0.948
22170 measured reflections
8040 independent reflections
6824 reflections with *I* > 2σ(*I*)
*R*_int_ = 0.023

#### Refinement

*R*[*F* ^2^ > 2σ(*F* ^2^)] = 0.040
*wR*(*F* ^2^) = 0.129
*S* = 1.08
8040 reflections
312 parameters
H-atom parameters constrained
Δρ_max_ = 0.71 e Å^−3^
Δρ_min_ = −0.55 e Å^−3^

### 

Data collection: *APEX2* (Bruker, 2009 ▶); cell refinement: *SAINT* (Bruker, 2009 ▶); data reduction: *SAINT*; program(s) used to solve structure: *SHELXTL* (Sheldrick, 2008 ▶); program(s) used to refine structure: *SHELXTL*; molecular graphics: *SHELXTL*; software used to prepare material for publication: *SHELXTL* and *PLATON* (Spek, 2009 ▶).

## Supplementary Material

Crystal structure: contains datablocks global, I. DOI: 10.1107/S1600536810024785/ci5115sup1.cif

Structure factors: contains datablocks I. DOI: 10.1107/S1600536810024785/ci5115Isup2.hkl

Additional supplementary materials:  crystallographic information; 3D view; checkCIF report

## Figures and Tables

**Table 1:** Hydrogen-bond geometry (Å, °)

*D*—H⋯*A*	*D*—H	H⋯*A*	*D*⋯*A*	*D*—H⋯*A*
C16—H16*A*⋯N1^i^	0.96	2.58	3.5018 (19)	161
C20—H20*A*⋯N3^ii^	0.96	2.59	3.5148 (19)	161

Symmetry codes: (i) 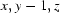; (ii) 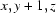.

**Fig. 1.**
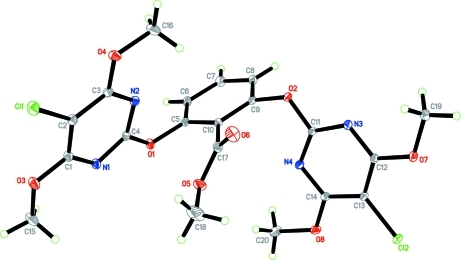
The molecular structure of the title compound, showing 30 % probability displacement ellipsoids for non-H atoms and the atom-numbering scheme.

**Fig. 2.**
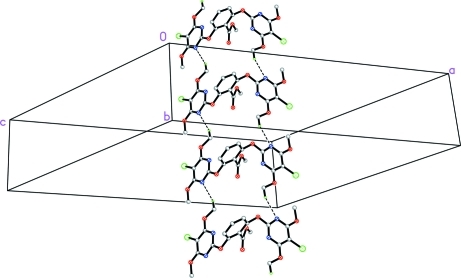
Part of the crystal structure, viewed along an arbitrary axis, showing a molecular ribbon. H atoms not involved in hydrogen bonds (dashed lines) have been omitted for clarity.

**Fig. 3.**
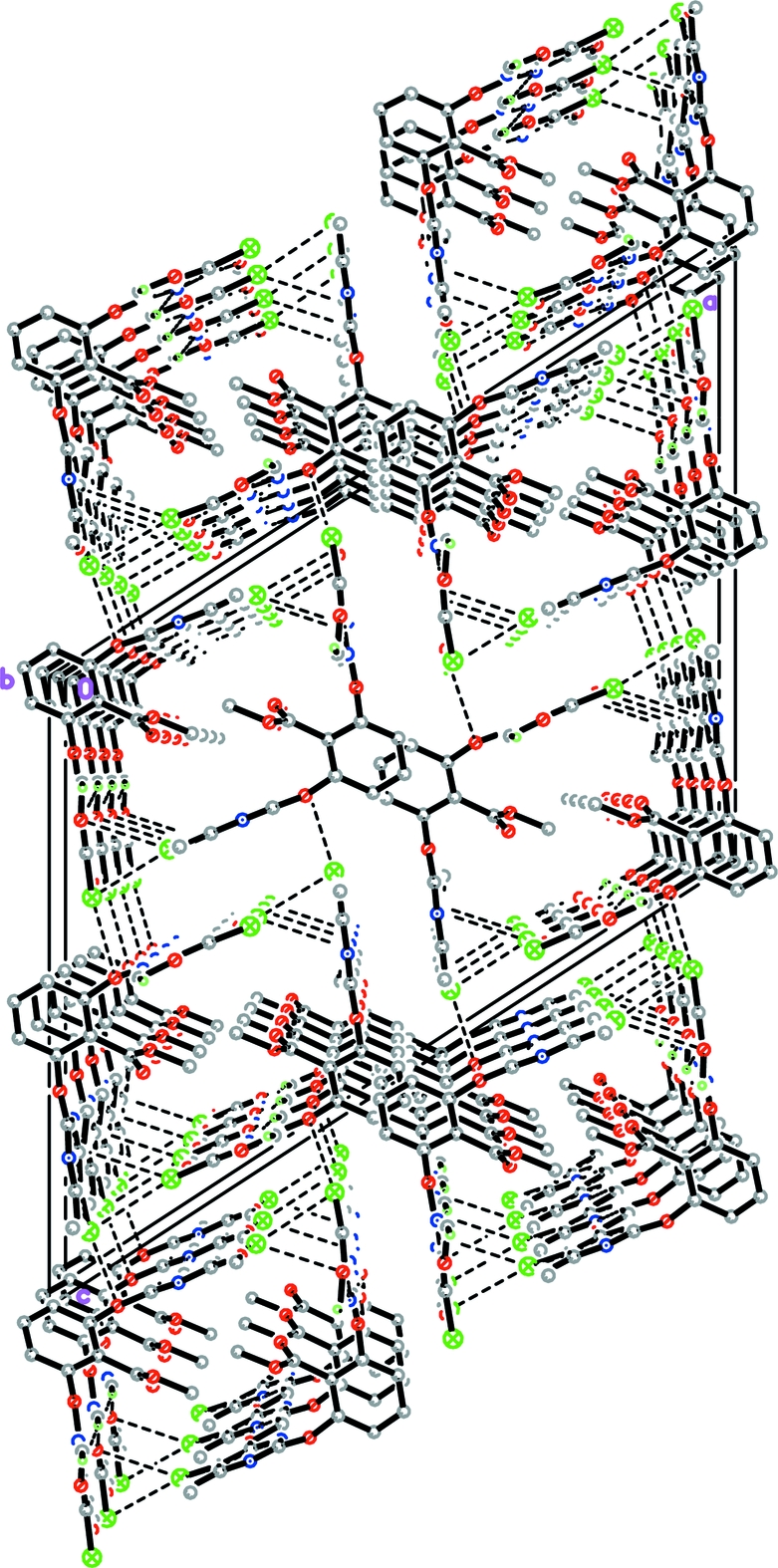
The crystal structure of the title compound, viewed along the b axis, showing two-dimensional networks parallel to the bc plane. H atoms not involved in intermolecular interactions (dashed lines) have been omitted for clarity.

## supplementary crystallographic information

### Comment

Methyl-2,6-bis[(5-bromo-4,6-dimethoxypyrimidin-2-yl)oxy]benzoate is a derivative of herbicide showing excellent herbicidal effects on annual and perennial weeds and high-safety crops, especially rice and wheat and is applied to paddy fields, ploughed fields and non-agricultural land (Koichiro *et al.*, 1988, 1998). Most sulphonylurea herbicides and all pyrimidinylbenzoate herbicides (He *et al.*, 2007) such as nicofulfuron, amidosulfuron, halopyrazosulfuron, ethoxysulfuron, pyriminobac-methyl and pyriftalid, possess 4,6-dimethoxypyrimidin-2-yl groups (Li *et al.*, 2006), while sulfometuron-methyl, a kind of sulfonylurea, contains 4,6-dimethylpyrimidin-2-yl groups, which suggests that the two disubstituted pyrimidin-2-yl groups possess high biological activity (Gerorge, 1983).

In the title compound (Fig. 1), the two pyrimidine rings (C1-C4/N1/N2 and C11-C14/N3/N4) are essentially planar, with maximum deviations of 0.011 (1) and 0.007 (1) Å, respectively, at atoms N1 and N4. The central phenyl ring is inclined at dihedral angles of 66.68 (5) and 71.91 (6)°, respectively, with respect to the C1-C4/N1/N2 and C11-C14/N3/N4 pyrimidine rings. The bond lengths and angles are consistent with a closely related structure (Li & Luo, 2006).

In the crystal structure, intermolecular C16—H16A···N1 and C20—H20A···N3 hydrogen bonds (Table 1) link neighbouring molecules into a ribbon-like structure containing *R*^2^_2_(26) ring motifs (Fig. 2, Bernstein *et al.*, 1995), along the *b* axis. The interesting features of the crystal structure are the intermolecular short Cl···Cl [Cl1···Cl2^iii^ = 3.4427 (6) Å; (iii) 1/2-x2, y-1/2, 1/2-z] and Cl···O [Cl1···O8^iii^ = 3.1750 (11) and Cl2···O1^iv^ = 3.1420 (9) Å; (iv) x, 2-y, z+1/2] interactions, which are shorter than the sum of the van der Waals radii of the relevant atoms, interconnecting the ribbons into two-dimensional networks parallel to the *bc* plane. The crystal structure is further stabilized by weak intermolecular π–π interactions [*Cg*1···*Cg*1^v^ = 3.4552 (8) Å; (v) -x, y, -z+1/2; *Cg*1 is the centroid of C11-C14/N3/N4 pyrimidine ring].

### Experimental

To a stirred solution of methyl-2,6-dihydroxybenzoate (0.50 g, 0.0026 mol) in acetonitrile (10 ml) was added potassium carbonate (1.00 g, 0.0070 mol) and 5-chloro-4,6-dimethoxy-2-(methylsulfonyl)pyrimidine (1.58 g, 0.0050 mol). The reaction mixture was heated to reflux for 4 h. Mass analysis showed completion of the reaction. The reaction mixture was filtered and the filtrate was concentrated. The residue was recrystallized using dichloromethane to obtain the title compound (yield: 67 %, m.p. 427–430 K).

### Refinement

All H atoms were placed in the calculated positions, with C–H = 0.93–0.96 Å, and refined using a riding model with *U*_iso_ = 1.2 or 1.5 *U*_eq_(C). The rotating group model was used for the methyl groups.

### Crystal data

**Table:**

C_20_H_18_Cl_2_N_4_O_8_	*F*(000) = 2112
*M**_r_* = 513.28	*D*_x_ = 1.523 Mg m^−^^3^
Monoclinic, *C*2/*c*	Mo *K*α radiation, λ = 0.71073 Å
Hall symbol: -C 2yc	Cell parameters from 9948 reflections
*a* = 29.354 (3) Å	θ = 2.7–32.6°
*b* = 8.0485 (8) Å	µ = 0.35 mm^−^^1^
*c* = 22.5923 (19) Å	*T* = 100 K
β = 123.014 (2)°	Block, colourless
*V* = 4475.7 (7) Å^3^	0.58 × 0.31 × 0.16 mm
*Z* = 8	

### Data collection

**Table:**

Bruker APEXII DUO CCD area-detector diffractometer	8040 independent reflections
Radiation source: fine-focus sealed tube	6824 reflections with *I* > 2σ(*I*)
graphite	*R*_int_ = 0.023
φ and ω scans	θ_max_ = 32.6°, θ_min_ = 1.7°
Absorption correction: multi-scan (*SADABS*; Bruker, 2009)	*h* = −44→43
*T*_min_ = 0.825, *T*_max_ = 0.948	*k* = −12→12
22170 measured reflections	*l* = −34→34

### Refinement

**Table:**

Refinement on *F*^2^	Primary atom site location: structure-invariant direct methods
Least-squares matrix: full	Secondary atom site location: difference Fourier map
*R*[*F*^2^ > 2σ(*F*^2^)] = 0.040	Hydrogen site location: inferred from neighbouring sites
*wR*(*F*^2^) = 0.129	H-atom parameters constrained
*S* = 1.08	*w* = 1/[σ^2^(*F*_o_^2^) + (0.0781*P*)^2^ + 1.9869*P*] where *P* = (*F*_o_^2^ + 2*F*_c_^2^)/3
8040 reflections	(Δ/σ)_max_ = 0.001
312 parameters	Δρ_max_ = 0.71 e Å^−^^3^
0 restraints	Δρ_min_ = −0.55 e Å^−^^3^

### Special details

**Table:**

Experimental. The crystal was placed in the cold stream of an Oxford Cryosystems Cobra open-flow nitrogen cryostat (Cosier & Glazer, 1986) operating at 100.0 (1)K.
Geometry. All esds (except the esd in the dihedral angle between two l.s. planes) are estimated using the full covariance matrix. The cell esds are taken into account individually in the estimation of esds in distances, angles and torsion angles; correlations between esds in cell parameters are only used when they are defined by crystal symmetry. An approximate (isotropic) treatment of cell esds is used for estimating esds involving l.s. planes.
Refinement. Refinement of F^2^ against ALL reflections. The weighted R-factor wR and goodness of fit S are based on F^2^, conventional R-factors R are based on F, with F set to zero for negative F^2^. The threshold expression of F^2^ > 2sigma(F^2^) is used only for calculating R-factors(gt) etc. and is not relevant to the choice of reflections for refinement. R-factors based on F^2^ are statistically about twice as large as those based on F, and R- factors based on ALL data will be even larger.

### Fractional atomic coordinates and isotropic or equivalent isotropic displacement parameters (Å^2^)

**Table:**

	*x*	*y*	*z*	*U*_iso_*/*U*_eq_	
Cl1	0.307309 (11)	0.57598 (4)	0.096234 (18)	0.02454 (8)	
Cl2	0.082300 (12)	0.91419 (4)	0.399331 (14)	0.01982 (8)	
O1	0.11797 (3)	0.91625 (11)	0.04367 (4)	0.01529 (16)	
O2	0.04987 (3)	0.57525 (11)	0.15715 (4)	0.01615 (16)	
O3	0.28912 (4)	0.93539 (12)	0.08837 (5)	0.02288 (19)	
O4	0.20796 (3)	0.42435 (11)	0.07368 (5)	0.01987 (17)	
O5	0.16660 (4)	0.88991 (14)	0.18631 (5)	0.0263 (2)	
O6	0.16179 (4)	0.62171 (15)	0.20959 (5)	0.0296 (2)	
O7	0.07442 (4)	0.55550 (12)	0.37357 (4)	0.01848 (17)	
O8	0.07019 (4)	1.06655 (11)	0.27317 (4)	0.01907 (17)	
N1	0.20277 (4)	0.92889 (13)	0.06785 (5)	0.01634 (18)	
N2	0.16079 (4)	0.66706 (13)	0.05757 (5)	0.01477 (17)	
N3	0.06228 (4)	0.56256 (13)	0.26376 (5)	0.01535 (18)	
N4	0.06122 (4)	0.82430 (13)	0.21320 (5)	0.01471 (17)	
C1	0.24667 (4)	0.84915 (16)	0.07879 (6)	0.0167 (2)	
C2	0.25047 (4)	0.67647 (16)	0.08112 (6)	0.0171 (2)	
C3	0.20559 (4)	0.58939 (15)	0.07043 (5)	0.0152 (2)	
C4	0.16281 (4)	0.83107 (15)	0.05732 (5)	0.01390 (19)	
C5	0.07753 (4)	0.82779 (14)	0.04464 (5)	0.01343 (18)	
C6	0.02637 (4)	0.83115 (16)	−0.01690 (5)	0.0172 (2)	
H6A	0.0207	0.8867	−0.0565	0.021*	
C7	−0.01650 (4)	0.75105 (17)	−0.01925 (6)	0.0206 (2)	
H7A	−0.0510	0.7533	−0.0605	0.025*	
C8	−0.00804 (4)	0.66766 (16)	0.03972 (6)	0.0179 (2)	
H8A	−0.0366	0.6133	0.0382	0.022*	
C9	0.04341 (4)	0.66658 (15)	0.10068 (5)	0.01393 (18)	
C10	0.08739 (4)	0.74658 (14)	0.10524 (5)	0.01335 (18)	
C11	0.05844 (4)	0.66055 (15)	0.21417 (5)	0.01376 (18)	
C12	0.06937 (4)	0.64190 (16)	0.32011 (5)	0.01463 (19)	
C13	0.07199 (4)	0.81463 (15)	0.32568 (5)	0.01496 (19)	
C14	0.06775 (4)	0.90169 (15)	0.26965 (5)	0.01476 (19)	
C15	0.28520 (6)	1.11370 (19)	0.08786 (9)	0.0296 (3)	
H15A	0.3151	1.1619	0.0877	0.044*	
H15B	0.2863	1.1497	0.1291	0.044*	
H15C	0.2516	1.1484	0.0464	0.044*	
C16	0.16177 (5)	0.33858 (18)	0.06581 (8)	0.0262 (3)	
H16A	0.1695	0.2218	0.0735	0.039*	
H16B	0.1306	0.3562	0.0190	0.039*	
H16C	0.1545	0.3807	0.0997	0.039*	
C17	0.14212 (4)	0.74193 (17)	0.17246 (6)	0.0187 (2)	
C18	0.22073 (7)	0.8981 (3)	0.24900 (9)	0.0473 (5)	
H18A	0.2353	1.0076	0.2534	0.071*	
H18B	0.2435	0.8182	0.2456	0.071*	
H18C	0.2194	0.8740	0.2897	0.071*	
C19	0.07186 (6)	0.37652 (18)	0.36714 (6)	0.0226 (2)	
H19A	0.0735	0.3285	0.4072	0.034*	
H19B	0.1019	0.3373	0.3653	0.034*	
H19C	0.0384	0.3449	0.3247	0.034*	
C20	0.07386 (7)	1.15252 (18)	0.21994 (7)	0.0286 (3)	
H20A	0.0771	1.2698	0.2292	0.043*	
H20B	0.0418	1.1311	0.1743	0.043*	
H20C	0.1052	1.1140	0.2209	0.043*	

### Atomic displacement parameters (Å^2^)

**Table:**

	*U*^11^	*U*^22^	*U*^33^	*U*^12^	*U*^13^	*U*^23^
Cl1	0.01660 (12)	0.02313 (16)	0.03475 (16)	0.00517 (10)	0.01454 (11)	0.00213 (12)
Cl2	0.02636 (14)	0.02056 (15)	0.01647 (12)	−0.00313 (10)	0.01420 (10)	−0.00502 (9)
O1	0.0144 (3)	0.0143 (4)	0.0208 (3)	0.0030 (3)	0.0119 (3)	0.0037 (3)
O2	0.0246 (4)	0.0139 (4)	0.0150 (3)	−0.0023 (3)	0.0141 (3)	−0.0023 (3)
O3	0.0179 (4)	0.0186 (4)	0.0352 (5)	0.0000 (3)	0.0164 (3)	0.0014 (4)
O4	0.0170 (3)	0.0131 (4)	0.0280 (4)	0.0012 (3)	0.0113 (3)	−0.0011 (3)
O5	0.0220 (4)	0.0305 (5)	0.0185 (4)	−0.0102 (4)	0.0060 (3)	−0.0027 (4)
O6	0.0222 (4)	0.0338 (6)	0.0234 (4)	0.0043 (4)	0.0064 (3)	0.0104 (4)
O7	0.0266 (4)	0.0176 (4)	0.0158 (3)	−0.0012 (3)	0.0144 (3)	0.0008 (3)
O8	0.0267 (4)	0.0131 (4)	0.0175 (3)	0.0004 (3)	0.0121 (3)	−0.0015 (3)
N1	0.0160 (4)	0.0151 (5)	0.0205 (4)	0.0010 (3)	0.0116 (3)	0.0012 (3)
N2	0.0148 (4)	0.0142 (4)	0.0162 (3)	0.0020 (3)	0.0090 (3)	0.0003 (3)
N3	0.0185 (4)	0.0154 (5)	0.0155 (4)	−0.0007 (3)	0.0114 (3)	−0.0009 (3)
N4	0.0179 (4)	0.0139 (4)	0.0139 (3)	0.0006 (3)	0.0097 (3)	−0.0008 (3)
C1	0.0146 (4)	0.0176 (5)	0.0190 (4)	−0.0001 (4)	0.0100 (3)	0.0005 (4)
C2	0.0141 (4)	0.0174 (5)	0.0206 (4)	0.0033 (4)	0.0100 (3)	0.0008 (4)
C3	0.0153 (4)	0.0142 (5)	0.0156 (4)	0.0023 (4)	0.0081 (3)	0.0002 (4)
C4	0.0135 (4)	0.0156 (5)	0.0140 (4)	0.0023 (4)	0.0083 (3)	0.0011 (4)
C5	0.0142 (4)	0.0135 (5)	0.0155 (4)	0.0013 (4)	0.0100 (3)	−0.0001 (4)
C6	0.0165 (4)	0.0212 (6)	0.0144 (4)	0.0016 (4)	0.0088 (3)	0.0019 (4)
C7	0.0154 (4)	0.0270 (7)	0.0167 (4)	−0.0003 (4)	0.0071 (3)	0.0010 (4)
C8	0.0162 (4)	0.0211 (6)	0.0182 (4)	−0.0024 (4)	0.0105 (4)	−0.0020 (4)
C9	0.0178 (4)	0.0133 (5)	0.0137 (4)	−0.0001 (4)	0.0105 (3)	−0.0011 (4)
C10	0.0144 (4)	0.0136 (5)	0.0130 (4)	0.0007 (3)	0.0081 (3)	−0.0009 (3)
C11	0.0146 (4)	0.0154 (5)	0.0136 (4)	−0.0007 (4)	0.0092 (3)	−0.0020 (4)
C12	0.0146 (4)	0.0177 (5)	0.0135 (4)	−0.0005 (4)	0.0089 (3)	0.0000 (4)
C13	0.0171 (4)	0.0161 (5)	0.0139 (4)	−0.0004 (4)	0.0099 (3)	−0.0025 (4)
C14	0.0153 (4)	0.0142 (5)	0.0149 (4)	0.0006 (4)	0.0083 (3)	−0.0013 (4)
C15	0.0243 (6)	0.0186 (6)	0.0490 (8)	−0.0024 (5)	0.0220 (6)	0.0008 (6)
C16	0.0207 (5)	0.0153 (6)	0.0407 (7)	−0.0013 (4)	0.0155 (5)	−0.0015 (5)
C17	0.0161 (4)	0.0242 (6)	0.0156 (4)	−0.0012 (4)	0.0085 (3)	0.0002 (4)
C18	0.0290 (7)	0.0602 (13)	0.0281 (7)	−0.0201 (8)	−0.0003 (6)	−0.0017 (7)
C19	0.0323 (6)	0.0183 (6)	0.0214 (5)	−0.0017 (5)	0.0173 (4)	0.0016 (5)
C20	0.0497 (8)	0.0162 (6)	0.0220 (5)	0.0018 (6)	0.0209 (5)	0.0020 (5)

### Geometric parameters (Å, °)

**Table:**

Cl1—C2	1.7126 (11)	C5—C6	1.3834 (14)
Cl2—C13	1.7187 (11)	C5—C10	1.3978 (14)
O1—C4	1.3621 (12)	C6—C7	1.3889 (16)
O1—C5	1.3944 (13)	C6—H6A	0.93
O2—C11	1.3569 (12)	C7—C8	1.3881 (16)
O2—C9	1.3929 (13)	C7—H7A	0.93
O3—C1	1.3370 (14)	C8—C9	1.3824 (14)
O3—C15	1.4393 (18)	C8—H8A	0.93
O4—C3	1.3301 (14)	C9—C10	1.3952 (14)
O4—C16	1.4436 (15)	C10—C17	1.4934 (15)
O5—C17	1.3370 (16)	C12—C13	1.3943 (17)
O5—C18	1.4432 (17)	C13—C14	1.3918 (15)
O6—C17	1.2023 (16)	C15—H15A	0.96
O7—C12	1.3301 (13)	C15—H15B	0.96
O7—C19	1.4457 (16)	C15—H15C	0.96
O8—C14	1.3288 (14)	C16—H16A	0.96
O8—C20	1.4415 (15)	C16—H16B	0.96
N1—C4	1.3224 (14)	C16—H16C	0.96
N1—C1	1.3365 (14)	C18—H18A	0.96
N2—C4	1.3215 (16)	C18—H18B	0.96
N2—C3	1.3372 (14)	C18—H18C	0.96
N3—C11	1.3238 (14)	C19—H19A	0.96
N3—C12	1.3353 (13)	C19—H19B	0.96
N4—C11	1.3214 (16)	C19—H19C	0.96
N4—C14	1.3360 (13)	C20—H20A	0.96
C1—C2	1.3930 (18)	C20—H20B	0.96
C2—C3	1.3923 (15)	C20—H20C	0.96
			
C4—O1—C5	117.72 (9)	N3—C11—O2	112.86 (10)
C11—O2—C9	117.70 (9)	O7—C12—N3	119.87 (11)
C1—O3—C15	116.92 (10)	O7—C12—C13	117.78 (9)
C3—O4—C16	116.97 (9)	N3—C12—C13	122.35 (10)
C17—O5—C18	115.74 (13)	C14—C13—C12	116.54 (9)
C12—O7—C19	117.10 (9)	C14—C13—Cl2	121.81 (9)
C14—O8—C20	117.10 (9)	C12—C13—Cl2	121.61 (8)
C4—N1—C1	114.76 (11)	O8—C14—N4	119.85 (10)
C4—N2—C3	115.27 (9)	O8—C14—C13	118.24 (10)
C11—N3—C12	114.78 (10)	N4—C14—C13	121.92 (11)
C11—N4—C14	115.29 (9)	O3—C15—H15A	109.5
N1—C1—O3	120.02 (11)	O3—C15—H15B	109.5
N1—C1—C2	122.37 (10)	H15A—C15—H15B	109.5
O3—C1—C2	117.60 (10)	O3—C15—H15C	109.5
C3—C2—C1	116.54 (10)	H15A—C15—H15C	109.5
C3—C2—Cl1	121.59 (10)	H15B—C15—H15C	109.5
C1—C2—Cl1	121.87 (9)	O4—C16—H16A	109.5
O4—C3—N2	119.63 (10)	O4—C16—H16B	109.5
O4—C3—C2	118.50 (10)	H16A—C16—H16B	109.5
N2—C3—C2	121.87 (11)	O4—C16—H16C	109.5
N2—C4—N1	129.15 (10)	H16A—C16—H16C	109.5
N2—C4—O1	117.63 (9)	H16B—C16—H16C	109.5
N1—C4—O1	113.22 (10)	O6—C17—O5	124.13 (11)
C6—C5—O1	116.19 (9)	O6—C17—C10	124.84 (12)
C6—C5—C10	121.70 (9)	O5—C17—C10	111.03 (10)
O1—C5—C10	122.04 (9)	O5—C18—H18A	109.5
C5—C6—C7	119.62 (10)	O5—C18—H18B	109.5
C5—C6—H6A	120.2	H18A—C18—H18B	109.5
C7—C6—H6A	120.2	O5—C18—H18C	109.5
C8—C7—C6	120.21 (10)	H18A—C18—H18C	109.5
C8—C7—H7A	119.9	H18B—C18—H18C	109.5
C6—C7—H7A	119.9	O7—C19—H19A	109.5
C9—C8—C7	119.10 (10)	O7—C19—H19B	109.5
C9—C8—H8A	120.4	H19A—C19—H19B	109.5
C7—C8—H8A	120.4	O7—C19—H19C	109.5
C8—C9—O2	116.45 (9)	H19A—C19—H19C	109.5
C8—C9—C10	122.36 (10)	H19B—C19—H19C	109.5
O2—C9—C10	121.14 (9)	O8—C20—H20A	109.5
C9—C10—C5	117.01 (9)	O8—C20—H20B	109.5
C9—C10—C17	120.20 (9)	H20A—C20—H20B	109.5
C5—C10—C17	122.79 (9)	O8—C20—H20C	109.5
N4—C11—N3	129.10 (10)	H20A—C20—H20C	109.5
N4—C11—O2	118.04 (9)	H20B—C20—H20C	109.5
			
C4—N1—C1—O3	178.40 (10)	C8—C9—C10—C17	179.84 (11)
C4—N1—C1—C2	−1.96 (15)	O2—C9—C10—C17	−2.78 (16)
C15—O3—C1—N1	1.42 (16)	C6—C5—C10—C9	0.94 (16)
C15—O3—C1—C2	−178.24 (11)	O1—C5—C10—C9	177.93 (10)
N1—C1—C2—C3	0.85 (16)	C6—C5—C10—C17	−179.62 (11)
O3—C1—C2—C3	−179.50 (10)	O1—C5—C10—C17	−2.62 (17)
N1—C1—C2—Cl1	−179.21 (8)	C14—N4—C11—N3	−1.47 (16)
O3—C1—C2—Cl1	0.44 (15)	C14—N4—C11—O2	177.68 (9)
C16—O4—C3—N2	−2.55 (15)	C12—N3—C11—N4	0.79 (16)
C16—O4—C3—C2	177.03 (10)	C12—N3—C11—O2	−178.39 (9)
C4—N2—C3—O4	178.15 (10)	C9—O2—C11—N4	−1.34 (13)
C4—N2—C3—C2	−1.42 (14)	C9—O2—C11—N3	177.94 (9)
C1—C2—C3—O4	−178.62 (10)	C19—O7—C12—N3	0.01 (14)
Cl1—C2—C3—O4	1.43 (14)	C19—O7—C12—C13	−179.72 (10)
C1—C2—C3—N2	0.95 (15)	C11—N3—C12—O7	−179.16 (9)
Cl1—C2—C3—N2	−178.99 (8)	C11—N3—C12—C13	0.55 (14)
C3—N2—C4—N1	0.15 (16)	O7—C12—C13—C14	178.67 (9)
C3—N2—C4—O1	179.26 (9)	N3—C12—C13—C14	−1.04 (15)
C1—N1—C4—N2	1.52 (16)	O7—C12—C13—Cl2	0.85 (14)
C1—N1—C4—O1	−177.62 (9)	N3—C12—C13—Cl2	−178.87 (8)
C5—O1—C4—N2	12.29 (13)	C20—O8—C14—N4	−9.19 (15)
C5—O1—C4—N1	−168.46 (9)	C20—O8—C14—C13	170.99 (11)
C4—O1—C5—C6	−121.76 (11)	C11—N4—C14—O8	−179.01 (10)
C4—O1—C5—C10	61.09 (13)	C11—N4—C14—C13	0.81 (14)
O1—C5—C6—C7	−177.67 (11)	C12—C13—C14—O8	−179.86 (10)
C10—C5—C6—C7	−0.51 (18)	Cl2—C13—C14—O8	−2.04 (14)
C5—C6—C7—C8	−0.20 (19)	C12—C13—C14—N4	0.31 (15)
C6—C7—C8—C9	0.43 (19)	Cl2—C13—C14—N4	178.14 (8)
C7—C8—C9—O2	−177.46 (11)	C18—O5—C17—O6	2.3 (2)
C7—C8—C9—C10	0.03 (18)	C18—O5—C17—C10	−177.92 (13)
C11—O2—C9—C8	−110.10 (11)	C9—C10—C17—O6	41.16 (17)
C11—O2—C9—C10	72.38 (13)	C5—C10—C17—O6	−138.26 (13)
C8—C9—C10—C5	−0.70 (16)	C9—C10—C17—O5	−138.63 (11)
O2—C9—C10—C5	176.68 (10)	C5—C10—C17—O5	41.94 (15)

### Hydrogen-bond geometry (Å, °)

**Table:**

*D*—H···*A*	*D*—H	H···*A*	*D*···*A*	*D*—H···*A*
C16—H16A···N1^i^	0.96	2.58	3.5018 (19)	161
C20—H20A···N3^ii^	0.96	2.59	3.5148 (19)	161

Symmetry codes: (i) *x*, *y*−1, *z*; (ii) *x*, *y*+1, *z*.
